# Deep Venous Thrombosis after Ad26.COV2.S Vaccination in Adult Male

**DOI:** 10.1155/2021/7682655

**Published:** 2021-10-14

**Authors:** Habiba Hussain, Matthew Sehring, Sheryll Soriano

**Affiliations:** ^1^Department of Pulmonary and Critical Care Medicine Fellowship, University of Illinois College of Medicine at Peoria, IL, USA; ^2^Pulmonary and Critical Care Medicine, OSF St. Mary and OSF Saint Luke Medical Center, IL, USA; ^3^Department of Pulmonary and Critical Care Medicine, University of Illinois College of Medicine at Peoria, IL, USA

## Abstract

With extensive loss of life and well-being seen since the beginning of the SARS-CoV-2 pandemic, the initiation of vaccinations has come with enormous hope towards the end of this pandemic. Detailed discussions regarding the safety and efficacy of these vaccines led to their approval. With such success, there have also been reports of vaccine-associated adverse events—allergic reactions, anaphylaxis, immune thrombocytopenia, and thrombosis. We discuss and report the first case of a healthy young adult male developing extensive thrombosis, after receiving the Ad26.COV2.S (Johnson & Johnson/Janssen) vaccine.

## 1. Introduction

There have been over 147,000,000 cases of SARS-CoV-2 worldwide so far including over 3,000,000 deaths, leading to an insurmountable loss of life and well-being [1]. So far, over 900,000,000 doses of various vaccines have been administered worldwide [1]. Cases of thrombocytopenia following Pfizer-BioNTech (BNT162b2) and Moderna (mRNA-1273) vaccines while atypical thrombosis following ChAdOx1 nCoV-19 and Ad26.COV2.S have been reported. Around 15 cases of Ad26.COV2.S (Johnson & Johnson/Janssen) vaccine-associated thrombosis have been reported in women [2–5]. Of which, cerebral venous sinus thrombosis led to a temporary halt in its administration. Although the vaccine administration has now resumed, its resumption is included with handout warning of the risk of clots in women under 50 years [5, 6].

## 2. Case Description

A 21-year-old male without significant past medical history presented to the emergency department 8 days after receiving the Ad26.COV2.S vaccine, with complaints of left groin pain and inability to bear weight on the left leg. On initial evaluation, he was noted to have mild erythema and edema of the left leg with circumferential diameter 40 cm comparative to 38 cm of the right leg. He was recommended to follow supportive care with conservative approach and discharged home. A day later, he presented to the emergency department with worsening swelling of the left leg. On examination, left lower extremity edema was consistent with prior exam with new skin changes concerning for phlegmasia cerulea dolens. Lower extremity duplex showed absent blood flow in the left common femoral, profundal femoral, femoral, and calf veins due to occluding thrombus visualized from the iliac, femoral, popliteal to the left posterior tibial, and peroneal vein. Complete blood count including platelet count was normal. Coagulation panel included an elevated INR at 14.3, PTT at 120 sec, and decreased fibrinogen at 172 mg/dl. He was noted to be negative for heparin-dependent platelet antibody (PF4) enzyme-linked immune sorbent assay (ELISA) IgA/M/G and serotonin release assay. He also received hypercoagulable workup for Factor V Leiden mutation, Prothrombin gene, serum homocysteine, anti-phospholipid antibody panel, antithrombin III activity, and protein C&S activity which were all unremarkable for underlying hypercoagulable etiology. He required left lower extremity venography with pharmacomechanical thrombolysis/thrombectomy with venous thrombolysis and placement of 16 mm × 100 mm left common iliac venous stent. He was maintained on direct thrombin inhibitor with argatroban, which was transitioned to oral apixaban upon discharge from the hospital. Venous duplex in one week is to be repeated. He will continue to follow with Hematology Outpatient.

## 3. Discussion

Thrombosis-related adverse events noted following recent SARS-CoV-2 vaccination have been classified as Vaccine-Induced Immune Thrombotic Thrombocytopenia (VITT) [7]. These cases of thrombosis in cerebral venous sinuses and splanchnic/portal/hepatic veins seen with ChAdOx1 nCoV-19 vaccine (AstraZeneca) and Ad26.COV2.S (Johnson & Johnson/Janssen) have been in the presence of thrombocytopenia (median of 20,000 to 30,000/mm^3^) and low levels of fibrinogen and antibody to platelet factor 4 (PF4) identified by ELISA without known exposure to heparin [5–8]. Although the pathogenesis for VITT is not completely understood, such presentation resembles autoimmune/atypical heparin-induced thrombocytopenia. Another caveat among these rare adverse events is the development of these thrombotic complications specifically in women and of younger age group < 50 years of age, where possible association may be considered with concomitant use of estrogen replacement therapy and/oral contraceptives [6, 7]. While these raise questions towards adenoviral vector vaccine, we present an index case of thrombosis not meeting these set criteria, also to be noted in an adenoviral vector vaccine.

To our knowledge, there have been no reported cases of extensive deep venous thrombosis with any of the recently administered SARS-CoV-2 vaccines in healthy young males (as young as 21 years of age) without significant past medical or contributory medication use history. This patient not only presents a unique demographic group for development of such extensive thrombosis crossing gender distribution. It also highlights the absence of risk factors apart from administration of vaccine for the nature of this extensive thrombosis causing phlegmasia requiring pharmacomechanical thrombolysis. While the absence of underlying risk factors was proven with unremarkable hypercoagulable workup, the absence of thrombocytopenia and PF4 ELISA antibodies also negates the classification of characteristic VITT [8]. This presentation extends the discussion for a possibility of PF4 immune complex antibody to be acting as an association rather than contribution/causation in thrombus propagation in such cases.

With resumption of Ad26.COV2.S (Johnson & Johnson/Janssen) vaccination now including warning handout, due to benefits outweighing the risk of such extremely rare clots, there still remains the need for further investigation to better understand the pathogenesis of clot development in these various clinical distribution patterns, immune involvement, and adenoviral vector association.

## Data Availability

Data is available in the electronic medical record.
